# PARANOiD: Pipeline for Automated Read ANalysis of iCLIP Data

**DOI:** 10.1093/bioinformatics/btaf673

**Published:** 2025-12-23

**Authors:** Patrick Barth, Frank Förster, Sebastian Jaenicke, Fabienne Thelen, Oliver Rossbach, Friedemann Weber, Lyudmila Shalamova, Alexander Goesmann

**Affiliations:** Bioinformatics and Systems Biology, Justus Liebig University Giessen, 35392 Giessen, Germany; Bioinformatics and Systems Biology, Justus Liebig University Giessen, 35392 Giessen, Germany; Bioinformatics Core Facility, Justus Liebig University Giessen, 35392 Giessen, Germany; Bioinformatics and Systems Biology, Justus Liebig University Giessen, 35392 Giessen, Germany; ELIXIR Germany, Institute of Bio- and Geosciences (IBG-5)–Computational Metagenomics, Forschungszentrum Jülich GmbH, 52425 Jülich, Germany; Bioinformatics and Systems Biology, Justus Liebig University Giessen, 35392 Giessen, Germany; Institute of Biochemistry, Faculty of Biology and Chemistry (FB08), Justus Liebig University Giessen, 35392 Giessen, Germany; German Center for Infection Research (DZIF), Partner Site Giessen-Marburg-Langen, Justus Liebig University Giessen, 35392 Giessen, Germany; German Center for Infection Research (DZIF), Partner Site Giessen-Marburg-Langen, Justus Liebig University Giessen, 35392 Giessen, Germany; Bioinformatics and Systems Biology, Justus Liebig University Giessen, 35392 Giessen, Germany

## Abstract

**Motivation:**

RNA–protein interactions play essential roles in every living organism, with RNA transcription, processing, and translation being just a few examples. Therefore, determining the set of RNAs that are bound by individual RNA-binding proteins, as well as the precise location of the interaction, is crucial for biological understanding. CLIP (UV-cross-linking and immunoprecipitation) is a method developed to study these interactions. Several variations of the CLIP protocol have been developed, e.g. iCLIP (individual-nucleotide resolution CLIP), which offers nucleotide-precise resolution of the cross-linking event.

**Results:**

PARANOiD is a versatile software for fully automated analysis of iCLIP and iCLIP2 data. It contains all steps necessary for preprocessing, the determination of cross-link locations, and several additional steps, which can be used to detect specific characteristics, e.g. definite distances between cross-link events or identify binding motifs. Additionally, results are visualized as statistical plots for a quick overview and as standardized bioinformatics file formats, which can be used for further analysis steps.

**Availability and implementation:**

PARANOiD is published under the MIT license and is available from https://github.com/patrick-barth/PARANOiD. The documentation is available at https://paranoid.readthedocs.io/en/latest/index.html.

## 1 Introduction

The individual-nucleotide resolution CLIP (iCLIP) (König et al. 2010) method is a variant of UV-cross-linking immunoprecipitation (CLIP). Both rely on UV-induced covalent cross-linking of proteins to nearby RNAs. Via immunoprecipitation, the target protein and all linked RNAs are extracted. After several purification steps and adapter ligation, a reverse transcription (RT) is performed. In about 80% of cases, RT stalls at the polypeptide linked to the RNA (Sugimoto *et al.* 2012). iCLIP utilizes this to provide single-nucleotide resolution of the cross-link position. After RT, the cDNA is purified, sequencing adapters are added, and the library is amplified by PCR. Finally, the RNAs that were bound to the protein of interest in live cells are determined by Illumina Sequencing. In 2020, an improved version named iCLIP2 was introduced, offering simplified library preparations, reduced sample loss, and prolonged barcode sequences (Buchbender *et al.* 2020). For a successful analysis of iCLIP data, we introduce PARANOiD. The workflow incorporates all required steps for automated analysis and interpretation of iCLIP and iCLIP2 data and has already been successfully used to analyze the interaction between the nucleoprotein and the Rift Valley Fever Virus genome (Shalamova *et al.* 2024). PARANOiD is made available as a Nextflow (Di Tommaso *et al.* 2017) workflow that can be deployed via the command-line-interface (CLI).

Beyond iCLIP, several other CLIP-seq variants have been developed, including Photo-activatable ribonucleoside CLIP (PAR-CLIP) (Hafner *et al.* 2010) and enhanced CLIP (eCLIP) (Van Nostrand *et al.* 2016). For these methods, dedicated analysis workflows have been published, such as the pipeline by Jens (2016) for PAR-CLIP and Skipper (Xu *et al.* 2024), CLIP-Explorer (Heyl *et al.* 2020) or racoon_clip (Klostermann and Zarnack 2024) for eCLIP. While PARANOiD is tailored to iCLIP and iCLIP2, these references can serve as useful guidance for users working with alternative protocols.

## 2 Methods

PARANOiD supports fast and efficient data analysis, as tasks can be executed in parallel and distributed to high-performance clusters, greatly reducing the overall processing duration. For all individual steps, Docker containers are provided, which can be run via Docker, Podman, Singularity, and Apptainer. This guarantees consistent versions for all tools included in the analysis process, supporting the reproducibility and comparability of results.

### 2.1 Required input data

PARANOiD usage requires (i) iCLIP or iCLIP2 sequence data in FASTQ format, (ii) a reference genome in FASTA format, and (iii) a TSV file assigning barcode sequences to their respective experiments. For splicing-capable organisms, an additional annotation file can be provided in either GFF or GTF3 format.

### 2.2 Data processing steps within PARANOiD

**Figure 1.**
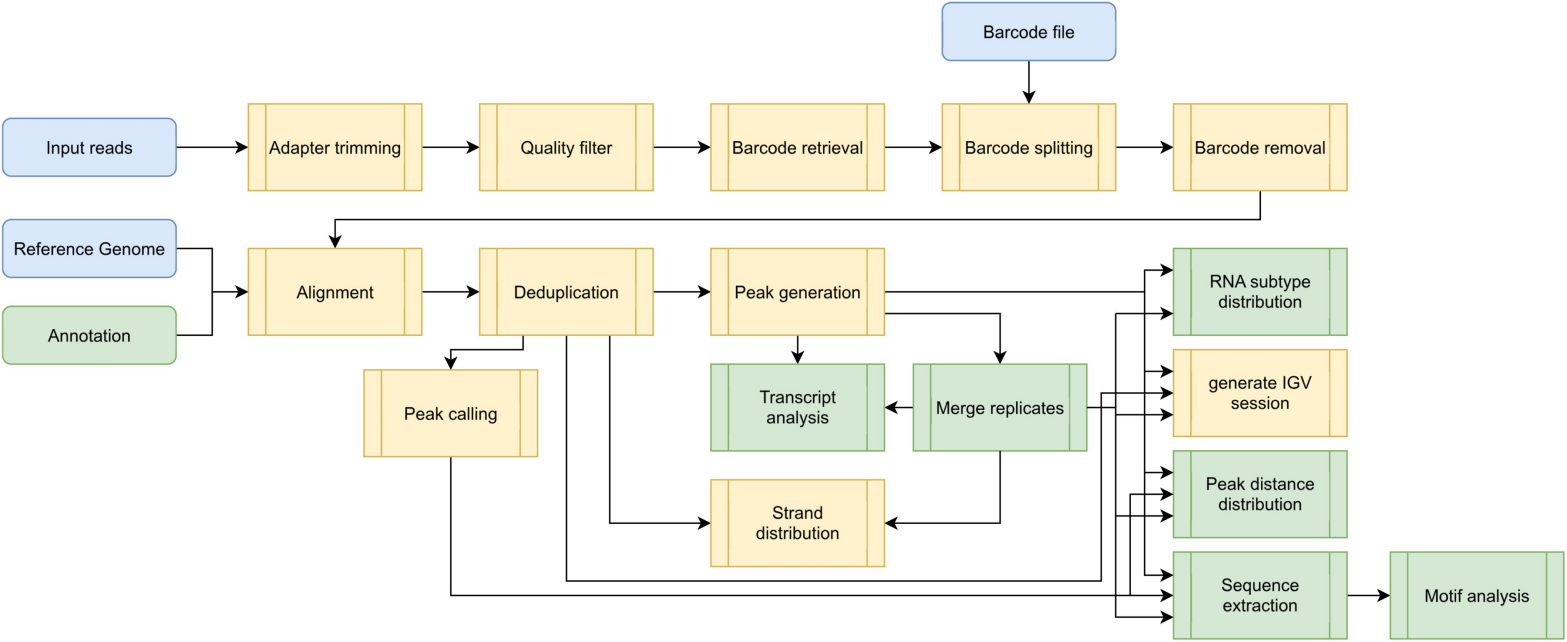
Overview of PARANOiD’s order of processes. Elements with rounded corners represent input files (only blue input files are required), while rectangular elements represent analysis processes. Green colored elements are optional and not part of the core Pipeline. A detailed description of all input parameters and processes can be found at https://paranoid.readthedocs.io/en/latest/.

PARANOiD provides analysis steps for quality control, removal of adapter sequences, trimming of low-quality and barcode sequences, and demultiplexing. Furthermore, several continuative analyses, e.g. peak calling, RNA subtype distribution, or sequence extraction, are included, which provide an in-depth view of the underlying data (Fig. 1). To provide a general overview to the user, a diverse array of statistics is collected during execution: Before and after preprocessing, read quality is surveyed using FastQC, and metrics provided by individual processing steps are collected and summarized by MultiQC (Ewels *et al.* 2016).

#### 2.2.1 Quality control and demultiplexing

Raw reads are first adapter trimmed using TrimGalore (https://zenodo.org/record/5127899), which relies upon cutadapt (Martin 2011). Afterwards, low-quality bases are removed via fastq_quality_filter from the FASTX toolkit (https://github.com/agordon/fastx_toolkit), and random barcode sequences are extracted via umi_tools (Smith *et al.* 2017). After the random barcode extraction, reads are assigned to their respective experiment using fastx_barcode_splitter from the FASTX toolkit, generating one FASTQ file per experiment, which will be processed in a parallel manner from thereon. The experimental barcode is removed using fastx_trimmer from the FASTX toolkit.

#### 2.2.2 Cross-link site identification

To identify cross-link sites, PARANOiD offers two different aligners. The default setting uses bowtie2 (Langmead and Salzberg 2012), with STAR (Dobin *et al.* 2013) being available as an alternative alignment tool. Afterwards, PCR duplicates are removed via umi_tools dedup. Since iCLIP experiments have different sources of background noise (Sugimoto *et al.* 2012, Jankowsky and Harris 2015), peak calling via PureCLIP (Krakau *et al.* 2017) is used by default, reducing the amount of false positive signal. Peak calling can be optionally omitted in which case cross-link sites are determined one nucleotide upstream of the alignments’ start position for reads aligned to the forward reference and one nucleotide downstream of the alignments’ end position for reads aligned to the reverse reference. The cross-link sites of all alignments are then summed up at their position using positive values for forward alignments and negative values for reverse alignments, which are exported to two distinct WIG files. If peak calling is performed, all subsequent analyses use confirmed peaks. Without peak calling, all further analyses have a variable cutoff applied to peaks, removing all peaks below a certain percentile (default: –percentile 90), calculated via numpy (Harris *et al.* 2020).

#### 2.2.3 Summarize replicates

The generation of several replicates per experiment is essential to draw statistically relevant conclusions. However, depicting all replicates can take up a lot of space, especially on posters where space is typically limited. To give users the possibility to publish the results generated by PARANOiD without having to depict every replicate, an optional merging of replicates is implemented. WIG files of replicates are summarized by calculating the arithmetic mean of the coverage for each position. If this option is chosen, all subsequent analyses are performed using the summarized experiments. Furthermore, a correlation analysis can be performed to provide a statistical measure of the similarity of replicates, using R (R. C. Team 2022). Correlation results are provided as CSV files and heatmaps generated using ggplot2 (Wickham 2016).

#### 2.2.4 Sequence extraction and motif discovery

As protein binding sites are often determined by protein-specific RNA motifs (Burd and Dreyfuss 1994), nucleotides surrounding detected cross-link sites can be used to determine these motifs. Therefore, an optional analysis step is provided that extracts a variable amount of nucleotides surrounding cross-link sites and performs motif analysis on extracted sequences using STREME (Bailey 2021). As the cross-link nucleotide itself tends to be biased toward uridines (Sugimoto *et al.* 2012), which can negatively impact and introduce experimental biases into a motif search, users can choose to omit the cross-link as well as neighboring nucleotides. Motif search results are provided in textual formats and as an HTML report.

#### 2.2.5 RNA subtype distribution

As certain proteins preferentially bind to specific RNAs, RNA types, or regions (Hirose *et al.* 2006), PARANOiD uses an optional analysis step that quantifies the cross-linking occurrences within specific RNA types via featureCounts (Liao *et al.* 2014). Results show the distribution of peaks over every RNA subtype present within the annotation file in textual form and as bar charts. Overlapping annotations will be shown as ambiguous.

#### 2.2.6 Peak distance distribution

Specific proteins were shown to cover target RNAs with periodic distance rather than via binding motifs, such as the hnRNP C protein (König *et al.* 2010) as well as the nucleocapsid proteins of diverse viruses (Egelman *et al.* 1989, Reguera *et al.* 2014). To address such periodic RNA–protein interactions, we provide an optional peak distance analysis, calculating the distance between individual cross-linking events. Results are provided as TSV files and line plots.

#### 2.2.7 Detailed transcript analysis

Transcripts often exhibit binding properties for specific proteins, and vice versa (Dubey *et al.* 2003), which can significantly impact the lifespan of the affected RNAs. Identifying transcripts that are likely to interact with a protein of interest can offer valuable insights into the molecular mechanisms and potential implications of these interactions. To facilitate this, an optional transcript analysis feature is implemented, allowing users to identify transcripts with the highest RNA–protein interaction counts. To utilize this analysis, users must use the –map_to_transcripts parameter and provide the transcripts instead of the reference genome for the –reference parameter. The top transcripts are delivered as a FASTA file, which can be visualized alongside the provided WIG files.

#### 2.2.8 Strand preference detection

Ambisense RNA viruses contain at least one genomic segment consisting of partly positive and partly negative polarity (Sabsay and Te Velthuis 2023). By observing the amount of forward and reverse aligned reads, insights about differing protein coverage of genomic and antigenomic fragments can be drawn. An overview of the amount of reads mapped to the forward and reverse reference is provided as output.

## 3 Conclusion

We provide PARANOiD, a novel modular Nextflow workflow to analyze iCLIP experiments. PARANOiD can be executed locally or via distributed compute infrastructures and uses containerized processes ensuring reproducibility due to version control. Supporting both iCLIP and iCLIP2 data, PARANOiD executes all processes necessary to perform an iCLIP data analysis, while offering diverse optional analysis steps. PARANOiD is independent of any specific species and also allows arbitrary sequences to be analyzed.

Output is provided in a variety of established bioinformatics file formats, supporting visualization and evaluation of analysis results. The generated IGV (Integrated Genomics Viewer) (Robinson *et al.* 2011) session file can be inserted directly into the IGV, automatically importing the reference file, together with generated tracks and if present, the annotation file.

PARANOiD greatly benefits from the modular architecture of workflow-based approaches; therefore, it is effortlessly possible to adapt PARANOiD to future developments.

## Data Availability

PARANOiD is published under the MIT license; it is implemented in Nextflow, R and Python 3; the source code is available at https://github.com/patrick-barth/PARANOID. Online documentation can be found at https://paranoid.readthedocs.io/en/latest/. We welcome general feedback, bug reports and enhancement suggestions. Use of tools from the MEME suite (i.e. STREME) is restricted to non-commercial purposes. If commercial exploitation of the motif discovery step within PARANOiD is planned, please adhere to the corresponding copyright (https://meme-suite.org/meme/doc/copyright.html).

## References

[btaf673-B1] Bailey TL. Streme: accurate and versatile sequence motif discovery. Bioinformatics 2021;37:2834–40. 10.1093/bioinformatics/btab203 33760053 PMC8479671

[btaf673-B2] Buchbender A , Mutter H, Sutandy FXR et al Improved library preparation with the new iCLIP2 protocol. Methods 2020;178:33–48. 31610236 10.1016/j.ymeth.2019.10.003

[btaf673-B3] Burd CG , Dreyfuss G. Conserved structures and diversity of functions of RNA-binding proteins. Science 1994;265:615–21. 10.1126/science.8036511 8036511

[btaf673-B4] Di Tommaso P , Chatzou M, Floden EW et al Nextflow enables reproducible computational workflows. Nat Biotechnol 2017;35:316–9. 10.1038/nbt.3820 28398311

[btaf673-B5] Dobin A , Davis CA, Schlesinger F et al Star: ultrafast universal RNA-seq aligner. Bioinformatics 2013;29:15–21. 10.1093/bioinformatics/bts635 23104886 PMC3530905

[btaf673-B6] Dubey AK , Baker CS, Suzuki K et al Csra regulates translation of the *Escherichia coli* carbon starvation gene, csta, by blocking ribosome access to the csta transcript. J Bacteriol 2003;185:4450–60. 10.1128/jb.185.15.4450-4460.2003 12867454 PMC165747

[btaf673-B7] Egelman EH , Wu SS, Amrein M et al The sendai virus nucleocapsid exists in at least four different helical states. J Virol 1989;63:2233–43. 10.1128/jvi.63.5.2233-2243.1989 2539515 PMC250641

[btaf673-B8] Ewels P , Magnusson M, Lundin S et al Multiqc: summarize analysis results for multiple tools and samples in a single report. Bioinformatics 2016;32:3047–8. 10.1093/bioinformatics/btw354 27312411 PMC5039924

[btaf673-B9] Hafner M , Landthaler M, Burger L Jr, et al Transcriptome-wide identification of RNA-binding protein and microRNA target sites by PAR-CLIP. Cell 2010;141:129–41. 20371350 10.1016/j.cell.2010.03.009 PMC2861495

[btaf673-B10] Harris CR , Millman KJ, van der Walt SJ et al Array programming with numpy. Nature 2020;585:357–62. 10.1038/s41586-020-2649-2 32939066 PMC7759461

[btaf673-B11] Heyl F , Maticzka D, Uhl M et al Galaxy CLIP-explorer: A web server for CLIP-seq data analysis. GigasScience 2020. 9:1–11. 10.1093/gigascience/giaa108 PMC7657819 33179042

[btaf673-B12] Hirose T , Ideue T, Nagai M et al A spliceosomal intron binding protein, ibp160, links position-dependent assembly of intron-encoded box C/D SNORNP to pre-mRNA splicing. Mol Cell 2006;23:673–84. 10.1016/j.molcel.2006.07.011 16949364

[btaf673-B13] Jankowsky E , Harris ME. Specificity and nonspecificity in RNA–protein interactions. Nat Rev Mol Cell Biol 2015;16:533–44. 10.1038/nrm4032 26285679 PMC4744649

[btaf673-B14] Jens M. A pipeline for PAR-CLIP data analysis. Methods Mol Biol 2016;1358:197–207. 26463385 10.1007/978-1-4939-3067-8_12

[btaf673-B15] Klostermann M , Zarnack K. Racoon_clip-a complete pipeline for single-nucleotide analyses of iCLIP and eCLIP data. Bioinform Adv 2024;4:vbae084. 38948010 10.1093/bioadv/vbae084 PMC11213630

[btaf673-B16] König J , Zarnack K, Rot G et al iCLIP reveals the function of hnRNP particles in splicing at individual nucleotide resolution. Nat Struct Mol Biol 2010;17:909–15. 10.1038/nsmb.1838 20601959 PMC3000544

[btaf673-B17] Krakau S , Richard H, Marsico A. Pureclip: capturing target-specific protein–RNA interaction footprints from single-nucleotide clip-seq data. Genome Biol 2017;18:240. 10.1186/s13059-017-1364-2 29284540 PMC5746957

[btaf673-B18] Langmead B , Salzberg SL. Fast gapped-read alignment with bowtie 2. Nat Methods 2012;9:357–9. 10.1038/nmeth.1923 22388286 PMC3322381

[btaf673-B19] Liao Y , Smyth GK, Shi W. featurecounts: an efficient general purpose program for assigning sequence reads to genomic features. Bioinformatics 2014;30:923–30. 10.1093/bioinformatics/btt656 24227677

[btaf673-B20] Martin M. Cutadapt removes adapter sequences from high-throughput sequencing reads. EMBnet J 2011;17:10. 10.14806/ej.17.1.200

[btaf673-B21] Reguera J , Cusack S, Kolakofsky D. Segmented negative strand rna virus nucleoprotein structure. Curr Opin Virol 2014;5:7–15. 10.1016/j.coviro.2014.01.003 24486721

[btaf673-B22] Robinson JT , Thorvaldsdóttir H, Winckler W et al Integrative genomics viewer. Nat Biotechnol 2011;29:24–6. 10.1038/nbt.1754 21221095 PMC3346182

[btaf673-B23] Sabsay KR , Te Velthuis AJW. Negative and ambisense RNA virus ribonucleocapsids: more than protective armor. Microbiol Mol Biol Rev 2023;87:e0008223. 37750733 10.1128/mmbr.00082-23 PMC10732063

[btaf673-B24] Shalamova L , Barth P, Pickin MJ et al Nucleocapsids of the rift valley fever virus ambisense S segment contain an exposed RNA element in the center that overlaps with the intergenic region. Nat Commun 2024;15:7602. 39217162 10.1038/s41467-024-52058-2 PMC11365940

[btaf673-B25] Smith T , Heger A, Sudbery I. Umi-tools: modeling sequencing errors in unique molecular identifiers to improve quantification accuracy. Genome Res 2017;27:491–9. 10.1101/gr.209601.116 28100584 PMC5340976

[btaf673-B26] Sugimoto Y , König J, Hussain S et al Analysis of CLIP and iCLIP methods for nucleotide-resolution studies of protein–RNA interactions. Genome Biol 2012;13:R67. 10.1186/gb-2012-13-8-r67 22863408 PMC4053741

[btaf673-B27] R. C. Team. R: A Language and Environment for Statistical Computing. Vienna, Austria: R Foundation for Statistical Computing, 2022. https://www.R-project.org/

[btaf673-B28] Van Nostrand EL , Pratt GA, Shishkin AA et al Robust transcriptome-wide discovery of RNA-binding protein binding sites with enhanced CLIP (eCLIP). Nat Methods 2016;13:508–14. 27018577 10.1038/nmeth.3810 PMC4887338

[btaf673-B29] Wickham H. ggplot2: Elegant Graphics for Data Analysis. New York: Springer-Verlag, 2016. https://ggplot2.tidyverse.org

[btaf673-B30] Xu S , Nguyen GG, Naritomi JT et al Protocol to process crosslinking and immunoprecipitation data into annotated binding sites. STAR Protoc 2024;5:103040. 38669139 10.1016/j.xpro.2024.103040 PMC11066461

